# Effect of paternalistic leadership on Chinese youth elite athletes’ satisfaction: Resilience as a moderator

**DOI:** 10.3389/fpsyg.2022.1008163

**Published:** 2022-09-29

**Authors:** Pan Liu, Sitan Li, Qi Zhang, Xiumei Zhang, Lingling Guo, Juan Li

**Affiliations:** ^1^Beijing Institute of Petrochemical Technology, Beijing, China; ^2^Beijing Academy of Safety Engineering and Technology, Beijing, China; ^3^Moody College of Communication, The University of Texas at Austin, Austin, TX, United States; ^4^School of Health Care Security, Shandong First Medical University, Shandong, China; ^5^School of Physical Education, Wuhan Sports University, Wuhan, China

**Keywords:** satisfaction, youth athletes, mental resilience, paternalistic leadership, football schools

## Abstract

This study extended the research on the relationship between youth elite athletes’ satisfaction and coaches’ paternalistic leadership by identifying athletes’ resilience as a moderator. A total of 221 youth elite football (i.e., soccer) players aged 13–19 years old who are students of a Chinese professional football boarding school participated in a questionnaire survey. The study found no correlation between the three dimensions of coaches’ paternalistic leadership (authoritative leadership, benevolent leadership, and moral leadership) and the youth athletes’ satisfaction. The results also showed that the interaction of resilience and moral leadership affects the youth elite athletes’ satisfaction, whereas resilience does not play a moderating role in the relationship between authoritative leadership or benevolent leadership and satisfaction. As the results of the study are different from those of other scholars, they may reveal the uniqueness of youth elite football players in boarding schools. This study further analyzed the possible reasons for this result and prospected (or indicated) the theoretical and practical implications of these findings. Based on the conclusion, the study recommended that youth elite football schools should pay attention to the results of scientific research in leadership styles and apply them to practice in the future.

## Introduction

In China, professional football schools offer elite training programs different from campus football. In 2019, about 5,000 youth football players were studying in professional football boarding schools. These youth players are the key and core strength of the development of Chinese football and the source of Chinese football reserve talents. For example, the Shandong Luneng Taishan Football School, established in July 1999, has seen more than 230 players joining national football teams at all levels, and more than 260 players joining professional clubs in prestigious leagues such as the Chinese Super League, Chinese First League, and Chinese Second Class League (The Chinese Football Association, 2019). The school has won 70 national U13-U19 youth football matches and 8 international youth football tournaments (The Chinese Football Association, 2019).

The youth athletes studying in football boarding schools are typically 13–19 years old and are receiving unified national compulsory education. The elite athletes in these boarding schools are strictly selected and, it is the family’s honor to have a child accepted to be part of a Chinese Football Boarding School. Dropping out of a school or not meeting the expectations might be considered a dishonor to the athlete as well as the family. Therefore, athletes in a Chinese Football Boarding School would be expected to accept the way the program is operated to a certain degree. For youth players attending boarding schools, the role of the parents is weakened, and their coaches and teachers are responsible for their training and other aspects of life. For the safety, learning, training, education, and growth of young athletes, each coach and teacher is responsible for managing 20–30 athletes. Therefore, it is difficult for them to observe the athletes’ psychological changes and be a part of all aspects of the athletes’ life. In addition, youth athletes are energetic, psychologically sensitive, and have limited emotional management capabilities (Zhang, 2019). At this time, they need to independently deal with their relationships with coaches, peers, and families, as well as manage the relationship between their life, training, and learning. Therefore, for their personal safety considerations, and also for their physical and mental health, various professional football schools have successively implemented boarding school management systems.

Although the boarding school management system in China has not been empirically researched yet, in practice, the management staff believes that introducing it as an intervention strategy (school management regulations and teaching measures) can affect youth athletes’ cognition, behavior, and psychology. However, whether it affects the youth athletes’ perceptions of themselves, their teams, and their coaches’ leadership requires research to provide empirical evidence. Moreover, China’s current sports psychology and management research related to football is concentrated on college student-athletes and campus football, and only a small portion of the literature is devoted to professional football (Gong et al., 2017; Li et al., 2018; Gu, 2019). Particularly, research on youth athletes and coaches of professional football schools remains scarce. Therefore, due to the importance of this group for the development of Chinese professional football, it deserves research attention.

### Athlete satisfaction

Athlete satisfaction has been defined as “a positive affective state resulting from a complex evaluation of the structures, processes, and outcomes associated with the athletic experience” (Chelladurai and Riemer, 1997, p. 135). Due to the randomness of sports results, the satisfaction of athletes is the main outcome variable and it is considered an indicator of coaches’ leadership (Chelladurai and Riemer, 1997) that can be obtained without analyzing sports performance big data. Furthermore, in sports psychology research, athlete satisfaction is also recognized as a very important variable (Burns et al., 2012).

However, the management of professional football schools and its relationship with the satisfaction of youth football players has received less attention. In the Shandong Luneng Taishan Football Schools’ internal evaluation systems for players, evaluation is one-dimensional, and the combination of coaches and teachers’ evaluations of youth athletes constitutes the final evaluation score. In the management routines of schools, youth athletes’ satisfaction with teachers’ work is also measured, but their satisfaction with self-performance and team performance is not measured (due to school regulations). To fill in this gap, this research has paid attention to the youth football players’ satisfaction with self-performance and team performance.

### Paternalistic leadership and athlete satisfaction

In recent years, research on paternalistic leadership has gone from theoretical (Farh and Cheng, 2000) to empirical (e.g., Cheng et al., 2002; Pellegrini and Scandura, 2006; Pellegrini et al., 2010; Fu et al., 2019). Paternalistic leadership is defined as a managerial approach that is based on strong discipline and authority combined with fatherly benevolence and moral integrity (Farh and Cheng, 2000). It can be divided into three dimensions: authoritarian, benevolent, and moral paternalistic leadership styles (Farh and Cheng, 2000). Authoritarian refers to a situation in which leaders have absolute authority and control over their subordinates, and require their subordinates to obey their actions unconditionally (Cheng et al., 2004). Benevolence implies that leaders not only focus on the personal wellbeing of their subordinates, but also show concern for the family welfare of their subordinates (Cheng et al., 2004). Moral paternalistic leadership is considered to be a leader’s behavior that demonstrates superior personal virtues, self-discipline, and selflessness (Cheng et al., 2004). Paternalistic leadership was previously found to be related to employee satisfaction (Yan et al., 2008; Pellegrini et al., 2010). Moreover, Chen and Tsai (2005) found that paternalistic leadership is related to athletes’ emotional feelings and burnout. Chang et al. (2019) studied the cross-level influence of paternalistic leadership on athlete burnout. Yang’s (2007) research noted the influence of coaches’ paternalistic leadership on coach-athlete relationships. These studies on coaches’ paternalistic leadership have drawn attention to the role of this variable in sports. Therefore, one might ask: in the context of youth sports, how does this variable affect satisfaction?

Models of athletic leadership often make use of satisfaction as an outcome variable (Riemer and Chelladurai, 1998). For example, in the study conducted by Aoyagi et al. (2008), 281 college student-athletes were surveyed using the athlete satisfaction questionnaire (ASQ)’s 6 subscales, to reveal the relationship between leadership and satisfaction in sport. Further, their research found that the coach’s leadership style had a significant impact on athlete’s satisfaction (Ignacio et al., 2017). Similarly, coach’s paternalistic leadership was found to have a positive effect on satisfaction toward the performance of the Division I volleyball athletes (Wu, 2010). In Chinese professional football schools, the coach is the person who spends the most time with youth athletes (according to the statements provided by the school’s management). The coaches not only lead in training but also live in the player’s apartments. Therefore, this study aims to provide further evidence on whether the youth athletes’ satisfaction is affected by the coaches’ paternalistic leadership style.

### Athlete resilience

Athletes’ resilience is another crucial factor in the success of sports performance. Fletcher and Sarkar (2012) defined resilience as “the role of mental processes and behavior in promoting personal assets and protecting an individual from the potential negative effect of stressors” (p. 675). There is much overlap between the content reflected in this concept and the “adversity view” in traditional Chinese culture (Hu and Gan, 2008). Affected by dialectical thinking, the Chinese have an optimistic and dialectical attitude toward adversity and misfortune, considering adversity to be a test given by God (i.e., God intends to test whether you can bear adversity). Chinese culture also posits that misfortune “may be a good matter” (because it will bring good results in the future; Hu and Gan, 2008). In terms of coping with major pressure, Confucianism focuses on relying on oneself to solve difficulties, adopting the middle way in dealing with problems (i.e., “moderation is the key to a happy life”), and not expressing emotions excessively (Yan, 2005); On the other hand, Taoism—another cornerstone of Chinese culture—promotes flexibility in the face of difficulties (Young et al., 2005). Recent cross-cultural research on coping styles also indicated that there is indeed a uniqueness in the behavioral patterns of Chinese people by showing their simultaneous use of primary control (change) and secondary control (acceptance) strategies (Heppner et al., 2006), an optimistic view (Wong, 2002), and a collectivist approach (Heppner et al., 2006). These characteristics of Chinese people are likely to be reflected in the process of facing great pressure and adversity, and constitute a unique component of Chinese football school athletes’ psychological resilience (Hu and Gan, 2008).

Based on the research of Olympic champions, Fletcher and Sarkar (2012) developed a grounded theory of psychological resilience and optimal sports performance. According to this theory, the influence of psychological factors should be analyzed in conjunction with the specific stressors that athletes encountered and the background of the stress incurred (Fletcher and Sarkar, 2012). The boarding life of the youth athletes may constitute the background of their stress. Youth athletes also need to participate and receive coaches’ training and guidance, which may be another source of stress for them. They do everything according to this schedule, and may not have their own time or flexibility in time management, which may also cause stress. According to the aforementioned grounded theory of psychological resilience (Fletcher and Sarkar, 2012), these stressors undergo psychological transformations within the athlete’s cognitive range and ultimately promote the athlete’s optimal athletic performance.

In addition, scholars also highlighted that the adjustment of resilience is vital to the athlete’s performance (Galli and Gonzalez, 2015). There are also studies showing that athlete resilience is pivotal in improving football performance (Chacón et al., 2016). This study replaced the youth athletes’ sports performance variable with athlete satisfaction (specifically toward individual performance and team performance). Based on the above theories, previous research findings, and cultural considerations, this study puts forward the following hypotheses. It is worth noting that the research team intentionally did not make directional predictions to reduce the researcher bias.

Hypothesis 1: Coach’s authoritarian leadership affects youth athletes’ satisfaction.

Hypothesis 2: Coach’s benevolent leadership affects youth athletes’ satisfaction.

Hypothesis 3: Coach’s moral leadership affects .youth athletes’ satisfaction.

Hypothesis 4: Youth athletes’ resilience plays a moderating role in the relationship between authoritarian leadership and youth athletes’ satisfaction.

Hypothesis 5: Youth athletes’ resilience plays a moderating role in the relationship between benevolent leadership and youth athletes’ satisfaction.

Hypothesis 6: Youth athletes’ resilience plays a moderating role in the relationship between moral leadership and youth athletes’ satisfaction.

**FIGURE 1:**
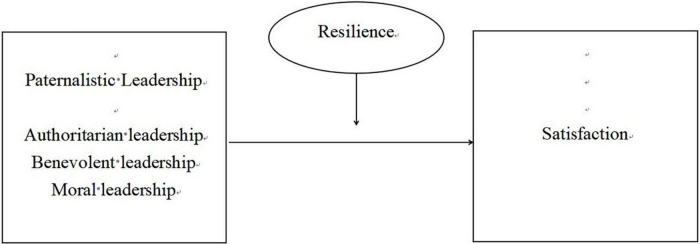
The research model.

The research model is depicted in Figure 1.

## Materials and methods

### Research design and statistical analysis

This research is a cross-sectional study that used, and it was approved and supported by the Academic Ethics Committee of Beijing Jiaotong University Informed consent from both the students and their parents/guardians was obtained. Participants were voluntary and not compensated for their participation. Furthermore, they were informed by the researchers of the purpose of the study, the anonymity of the questionnaire, and the voluntary nature of participating in this research. This study used Pearson’s α correlation analysis for descriptive statistics (see Supplementary Table 1), regression analysis to test the main effect and the moderating effect (Baron and Kenny, 1986), and the Bootstrap method proposed by Preacher and Hayes (2004) and Hayes (2013) to test the moderating effect. All analyses were completed using SPSSAU, an intelligent online statistical analysis platform (The SPSSAU Project, 2020) and SPSS 25.0.

### Participants

The participants were 221 youth football players (males) from Shandong Luneng Taishan Football School. The students were aged 13–19 years old (*M*_*age*_ = 15.15 ± 1.56) with the youngest students in the fifth grade of elementary school and the oldest in the third grade of high school. After excluding surveys in which all items were given the same answer, the regular answer, and blank answer, it was obtained 190 completed questionnaires (86%).

### Procedure

The data was collected from September 2019 to December 2019. The researchers in this study participated in the process of distributing and collecting questionnaires. The collection time and location were not fixed, and the collection time of each questionnaire was different. The researchers spent 12 weeks collecting data. During this period, participants completed multiple scales, including a paternalistic leadership scale, resilience scale, and satisfaction scale for this study.

### Measure

The revised Chinese Youth Psychological Resilience Scale (Hu and Gan, 2008) was used in this study, which is in line with the Chinese cultural characteristics. The scale includes 15 items, and five following factors: target concentration (e.g., “My life has clear goals”), emotional control (e.g., “I can adjust my emotions in a short time”), positive cognition (e.g., “I think adversity can inspire people”), family support (e.g., “My parents respect my opinion”), and interpersonal assistance (e.g., “When I am in trouble, I will take the initiative to talk to others”). Studies have shown that the reliability of the Chinese Youth Psychological Resilience Scale is satisfactory and has matched the characteristics of qualified psychological measurement. Each item in the scale is measured by a five-point Likert scale, ranging from 1 (never) to 5 (always). The reliability indexes of the five factors of the scale are: 0.654, 0.676, 0.854, 0.716, and 0.757. The overall reliability index measured in this study was 0.84.

This study adopted the two subscales of the (ASQ; Riemer and Chelladurai, 1998; Li et al., 2020) to measure the satisfaction of youth athletes. It focuses on two dimensions: individual’s satisfaction with their own task performance (e.g., “The degree to which I have reached my performance goals during the season”) and individual’s satisfaction with the performance of their team’s performance (e.g., “The team will break the record this season”). Each item in the scale is measured by a five-point Likert scale ranging from 1 (never) to 5 (always). Research has shown that both subscales have good psychometric reliability. The Cronbach’s α measured in this study for satisfaction with own task performance was 0.81, and with team performance was 0.82.

As suggested by the original author of the paternalistic leadership scale, the study simplified and revised the scale to have a better fit with the research context. After the reliability testing and factor analysis screening, this study ended up with a total of 15 items, among which the authoritarian leadership dimension included four items (e.g., “When the coach guides my technique and concept, he adopts the tone of command”), and the benevolent leadership dimension included five items (e.g., “The coach cares about my personal life”), whereas the moral leadership dimension contained a total of six items (e.g., “The knowledge and experience of the coach is enough to guide me”). Each item was measured on a five-point Likert scale, ranging from 1 (never) to 5 (always). The Cronbach’s α for each dimension were as follows: authoritarian leadership 0.725, benevolent leadership 0.689, and moral leadership 0.764.

## Results

### Main effect analysis

For the results of descriptive statistics (see Table 1 and Supplementary Table 1). As for the main effect analysis, the study used authoritarian leadership, benevolent leadership, moral leadership, resilience as independent variables and satisfaction as the dependent variable for a linear regression analysis (Dao-de, 2000). It can be seen from Table 1 authoritarian leadership, benevolent leadership, moral leadership, resilience explained only 2.1% change in satisfaction and the results of the *F* test showed that the model was not a good fit (*F* = 0.981, *p* = 0.419, n.s.). Therefore, that means, that authoritarian leadership, benevolent leadership, and moral leadership did not have a significant influence on youth athletes’ satisfaction. That is, hypothesis 1, 2, 3 is not supported.

**TABLE 1:** Linear regression analysis results (*N* = 190).

	Unstandardized coefficient		Standardized coefficient	*t*	*p*	VIF	*R* ^2^	Δ*R*^2^	*F*
	*B*	Standard error	*Beta*						
APL	0.036	0.065	0.042	0.548	0.584	1.099	0.021	0	*F*(4, 185) = 0.981, *p* = 0.419
BPL	0.08	0.08	0.083	0.998	0.32	1.295			
MPL	0.027	0.11	0.021	0.249	0.804	1.367			
Resilience	0.137	0.1	0.102	1.368	0.173	1.041			

APL, authoritative leadership; BPL, benevolent leadership; MPL, moral leadership; Dependent variable: satisfaction; D-W, 1.737.

### Moderating effect

It is important to note that the independent variables and moderating variables in this study were centralized. Using the analysis method of Baron and Kenny (1986), the moderating effect was divided into three models. Model 1 included independent variables (authoritarian leadership). Model 2 added a moderating variable (resilience) on the basis of Model 1, and Model 3 added an interaction (authoritarian leadership * resilience) on the basis of Model 2.

The purpose of Model 1 was to study the influence of independent variables (authoritarian leadership) on dependent variables (satisfaction) without considering the interference of moderating variables (resilience). From Supplementary Table 2, it can be drawn that that the independent variable (authoritarian leadership) was not significant (*t* = 0.614, *p* = 0.540, n.s.). This means that without considering the influence of moderating variables (resilience), authoritarian leadership did not have a significant influence on satisfaction.

The moderating effect can be viewed in two ways: the first is to look at the significance of the change in *F* value from Model 2 to Model 3; the second is to look at the significance of the interaction items in Model 3 (Hayes and Matthes, 2009; Hayes, 2013). This study analyzes the moderating effect in a second way.

In terms of Model 3, Supplementary Table 2 shows that the interaction between authoritarian leadership and resilience was not significant (*t* = −0.415, *p* = 0.679, n.s.). And from Model 1, they can see that authoritarian leadership did not have an effect on satisfaction. Therefore, that means, that youth athletes’ resilience does not play a moderating role in the relationship between authoritarian leadership and youth athletes’ satisfaction. So, hypothesis 4 is not verified.

Then, the study analyzed the effect of benevolent leadership on satisfaction. Benevolent leadership as the independent variable was also analyzed, and showed no significance (*t* = 1.159, *p* = 0.248, n.s.). It can be seen from Supplementary Table 3 that the interaction of benevolent leadership and resilience in Model 6 did not show significance (*t* = −1.356, *p* = 0.177, n.s.). And from Model 4, it can be drawn that benevolent leadership did not affect satisfaction, so there was no moderating effect. Thus, that means, that youth athletes’ resilience did not play a moderating role in the relationship between benevolent leadership and youth athletes’ satisfaction. So, hypothesis 5 is not verified too.

The results showed no significant moderating effect of resilience in the relationship between satisfaction and authoritative and benevolent leadership and include corresponding details as Supplementary Tables 2, 3.

Next, the study verified the effect of moral leadership as the independent variable on the dependent variable (satisfaction). It can be seen from Table 2 that moral leadership was not significant (*t* = 0.742, *p* = 0.459, n.s.). Implying no significant impact on satisfaction without considering the effect of the moderating variable (resilience). Then, the study looked at the significance of the interaction items in Table 2, Model 9. It can be seen that the interaction of moral leadership and resilience was significant (*t* = −2.241, *p* = 0.026, n.s.). This means that when moral leadership affects satisfaction when the moderating variable (resilience) is at different levels, the impact amplitude has a significant difference. Thus, that means, that youth athletes’ resilience played a moderating role in the relationship between moral leadership and youth athletes’ satisfaction. Finally, Hypothesis 6 was verified.

**TABLE 2:** Moral leadership moderating effect analysis results (*N* = 190).

	Model 7				Model 8				Model 9			
	*B*	Standard error	*t*	*p*	*B*	Standard error	*t*	*p*	*B*	Standard error	*t*	*p*
MPL	0.07	0.094	0.742	0.459	0.065	0.094	0.69	0.491	0.021	0.095	0.22	0.826
Resilience					0.141	0.098	1.437	0.152	0.079	0.101	0.787	0.432
MPL*Resilience									−0.303	0.135	−2.241	0.026*
*R* ^2^	0.003				0.014				0.04			
Adjusted *R*^2^	−0.002				0.003				0.024			
*F*	*F*(1, 188) = 0.550, *p* = 0.459				*F*(2, 187) = 1.309, *p* = 0.273				*F*(3, 186) = 2.565, *p* = 0.056			
△*R*^2^	0.003				0.011				0.026			
△*F*	*F*(1, 188) = 0.550, *p* = 0.459				*F*(1, 187) = 2.065, *p* = 0.152				*F*(1, 186) = 5.020, *p* = 0.026			

MPL, moral leadership; Dependent variable: satisfaction; *p < 0.05.

**FIGURE 2:**
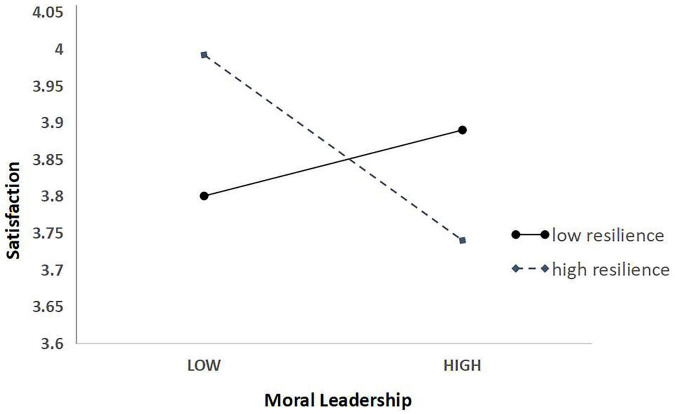
Moderating effect diagram.

The simple slope graph visually shows the difference in the magnitude (slope) of the influence of the independent variable on the dependent variable when the moderating variable is at different levels. When the moderating variable takes different levels, the influence variable (slope) difference of the independent variable on the dependent variable is the specific moderating effect. In other words, when resilience is higher, moral leadership has a greater impact on satisfaction, and when resilience is lower, moral leadership has a smaller impact on satisfaction. The slope analysis is shown in Supplementary Table 4, and the slope comparison chart is shown in Figure 2 depicts these results.

Then, the study used the Bootstrap method proposed by Preacher and Hayes (2004) and Hayes (2013) for verification. The analysis conclusion confirmed the above results.

## Discussion

This study measured and analyzed athlete satisfaction, the youth athletes’ resilience, and the coaches’ paternalistic leadership based on youth football players’ self-report. The results of the study showed that the coaches’ paternalistic leadership (all three dimensions) is not correlated with the youth athletes’ satisfaction. The results also show that the youth athletes’ resilience plays a moderating role in the relationship between moral leadership and satisfaction, whereas resilience does not play a moderating role in the relationship between authoritative leadership or benevolent leadership and satisfaction.

The first reason for the discrepancy between the findings of this research (in regard to the relationship between coaches’ paternalistic leadership and athlete satisfaction) and that suggested by previous literature is that other studies chose different subscales to measure this relationship (e.g., Riemer and Chelladurai, 1998; Aoyagi et al., 2008; Kao and Tsai, 2016). The second reason is the choice of the study sample, and age range. Most studies conducted their research on college athletes or adult professional elite athletes. Adolescents significantly differ from young college students or adults in terms of their physical and psychological developmental stages, and these differences might have affected the study results (Dorn and Biro, 2011). Therefore, it is plausible that in groups of youth athletes, findings that are different from the results based on other age groups may appear. The third reason is the particularity of the environment in which the research was conducted. In a nutshell, under the boarding school’s management system, the satisfaction of youth athletes did not show a correlation with coaches’ paternalistic leadership, thus other variables might be more consequential in determining satisfaction. To verify that, further research is needed.

Moreover, the study results showed that resilience plays a moderating role in the relationship between moral leadership and satisfaction. This finding is consistent with previous research that demonstrated the moderating effect of resilience in other fields of study (e.g., Zhou et al., 2017; Navarro et al., 2018; Fan and Lu, 2020; Zhang et al., 2020). However, in the present research, resilience played a moderating role only in the relationship between moral leadership and satisfaction, but not in the relationship between authoritative leadership or benevolent leadership and satisfaction. This should be viewed from the perspective of the definition of moral leadership. Moral leaders are not eager to be followed, but commit to serving subordinates, tend to help in developing subordinates’ skills, and are proficient in consulting skills (Farh and Cheng, 2000). Therefore, coaches may provide heuristic guidance education (such as imagination and autonomy) to athletes during training, rather than directly touching the ball or running a position demonstration. Athletes with stronger resilience may feel and understand that most of their personal and team achievements are the result of coaches’ inspiring guidance and services. Conversely, athletes with low resilience may feel that most of their athletic performance is the result of individual and team effort, and attribute the role of the coach to secondary. The resilience of youth is different from that of adults (Debold et al., 1999; Prince-Embury and Saklofske, 2012), and can improve through school education and coaching leadership (Kazak et al., 2010; Prince-Embury and Saklofske, 2012; Collishaw et al., 2016). Therefore, there are reasons to believe that it is meaningful to conduct further sports psychology research on resilience, especially aiming to implement resilience interventions in Chinese professional football boarding schools.

As Chinese professional football boarding schools and the management system they follow are critical to the development of football in China, it is essential to study the behavior of youth athletes and the variables associated with their satisfaction. Football school administrators base the practice on their own experience or learn from other countries’ management practice, but no in-depth scientific research has been conducted on the management system itself, nor has scientific research been applied to practice in this area (Yang et al., 2019). Therefore, our future research along this stream will continue to focus on coaching leadership, coach-athlete relationships, athlete satisfaction, athletes’ organizational citizenship behavior, trust, psychological resilience, stress, organizational environment, and team cohesion. If professional football schools in China can attribute the same importance to scientific research as youth football training institutions in Europe, and apply scientific research to management, it can result in great improvement in the quality of Chinese football. The limitation of this article also lies in the research on one representative professional football boarding school. There were no girls in the study and, if applicable, the use of similar questionnaires in participants of very different ages. In addition, due to the limitation of the number of students enrolled in the study, the sample that was somewhat reduced could influence the results. The reliability of the resilience scale is somewhat low in two factors (>0.70 is generally accepted). In the future, the research scope should be expanded to carrying out in-depth research in other professional football boarding schools, and other studies need to be done in similar programs in other cultures. Another limitation of this article is the use of cross-sectional data, and we will continue to follow up and validate the findings of this study in subsequent follow-up studies. In addition, there may be other influencing mechanisms in the relationship between paternalistic leadership and the youth football players’ satisfaction, waiting for future research to explore and verify.

## Conclusion

The results of this study did not provide support for the relationship between coach paternalistic leadership and athlete satisfaction, but they indicated the moderating effect of mental resilience on the relationship between the moral leadership dimension of coaches’ paternalistic leadership and athlete satisfaction. This result might have been driven by the characteristics of the present study sample and the environment, and this study provided insights into the youth athletes in Chinese professional football boarding schools, rather than adult or college student players as investigated in previous studies. This result is of great significance to the application of sports psychology and management in the field of youth football training in China. As the findings suggested, moral leadership is the best route for fostering resilience. Thus, it has important implications for future resilience interventions. To sum up, it is hoped that the contribution of this research will help deepen and further the limited understanding of athlete satisfaction, resilience, and coach leadership.

## Data availability statement

The raw data supporting the conclusions of this article will be made available by the authors, without undue reservation.

## Ethics statement

The studies involving human participants were reviewed and approved by Beijing Jiaotong University. Written informed consent to participate in this study was provided by the participants or their legal guardian/next of kin.

## Author contributions

JL made substantial contributions to conception of the work and wrote the manuscript. PL made contributions to the supervision and analysis of data for work. In the process of revision, SL and LG made contributions to interpretation of data. QZ and XZ revised the manuscript critically for important intellectual content.
