# Supplementary material for: Effect of paternalistic leadership on Chinese youth elite athletes’ satisfaction: Resilience as a moderator
Source: Front Psychol. 2022 Sep 29;13:1008163. doi: 10.3389/fpsyg.2022.1008163 (PMC9557739; doi:10.3389/fpsyg.2022.1008163)
Supplement: Supplementary file 1 [file Table_1.DOCX]

Supplementary Table 1

*Results of Descriptive Statistics (N= 190)*

|  | M | SD | APL | BPL | MPL | Satisfaction | Resilience |
| --- | --- | --- | --- | --- | --- | --- | --- |
| APL | 2.747 | 0.895 | 1 |  |  |  |  |
| BPL | 3.664 | 0.787 | -0.11 | 1 |  |  |  |
| MPL | 4.231 | 0.59 | -0.241** | 0.474** | 1 |  |  |
| Satisfaction | 3.841 | 0.761 | 0.045 | 0.084 | 0.054 | 1 |  |
| Resilience | 4.114 | 0.564 | 0.169* | -0.038 | 0.037 | 0.106 | 1 |

*Note.* M = mean; SD = standard deviation; APL = authoritative leadership; BPL = benevolent leadership; MPL = moral leadership; **p < .01; *p < .05
