# Supplementary material for: Effect of paternalistic leadership on Chinese youth elite athletes’ satisfaction: Resilience as a moderator
Source: Front Psychol. 2022 Sep 29;13:1008163. doi: 10.3389/fpsyg.2022.1008163 (PMC9557739; doi:10.3389/fpsyg.2022.1008163)
Supplement: Supplementary file 2 [file Table_2.DOCX]

Supplementary Table 2

*Authoritative Leadership Moderating Effect Analysis Results (N = 190)*

|  | Model 1 | | | | Model 2 | | | | Model 3 | | | |
| --- | --- | --- | --- | --- | --- | --- | --- | --- | --- | --- | --- | --- |
|  | *B* | Standard error | *t* | *p* | *B* | Standard error | *t* | *p* | *B* | Standard error | *t* | *p* |
| APL | .038 | .062 | .614 | .54 | .023 | .063 | 0.374 | .709 | .026 | .063 | .409 | .683 |
| Resilience |  |  |  |  | .137 | .099 | 1.378 | .17 | .132 | .1 | 1.317 | .19 |
| APL*Resilience |  |  |  |  |  |  |  |  | -.047 | .112 | -.415 | .679 |
| *R* ² | 0.002 | | | | .012 | | | | .013 | | | |
| Adjusted *R* ² | -0.003 | | | | .001 | | | | -.003 | | | |
| *F* | *F*(1,188)=.377, *p*=.540 | | | | *F*(2,187)=1.139, *p*=0.322 | | | | *F*(3,186)=.813, *p*=.488 | | | |
| △*R* ² | .002 | | | | .01 | | | | .001 | | | |
| △*F* | *F*(1,188)=.377,*p*=.540 | | | | *F*(1,187)=1.899, *p*=.170 | | | | *F*(1,186)=.172, *p*=.679 | | | |

*Note.* APL = authoritative leadership; Dependent variable: satisfaction; ***p* < .01; **p* < .05
