# Supplementary material for: Effect of paternalistic leadership on Chinese youth elite athletes’ satisfaction: Resilience as a moderator
Source: Front Psychol. 2022 Sep 29;13:1008163. doi: 10.3389/fpsyg.2022.1008163 (PMC9557739; doi:10.3389/fpsyg.2022.1008163)
Supplement: Supplementary file 3 [file Table_3.DOCX]

Supplementary Table 3

*Benevolent Leadership Moderating Effect Analysis Results (N = 190)*

|  | Model 4 | | | | Model 5 | | | | Model 6 | | | |
| --- | --- | --- | --- | --- | --- | --- | --- | --- | --- | --- | --- | --- |
|  | *B* | Standard error | *t* | *p* | *B* | Standard error | *t* | *p* | *B* | Standard error | *t* | *p* |
| BPL | .081 | .07 | 1.159 | .248 | .085 | .07 | 1.219 | .224 | .106 | .072 | 1.486 | .139 |
| Resilience |  |  |  |  | .148 | .098 | 1.512 | .132 | .118 | .1 | 1.175 | .241 |
| BPL*Resilience |  |  |  |  |  |  |  |  | -.169 | .125 | -1.356 | 0.177 |
| *R ²* | .007 | | | | .019 | | | | .029 | | | |
| Adjust *R ²* | .002 | | | | .009 | | | | .013 | | | |
| *F* | *F*(1,188)=1.343, *p*=.248 | | | | *F*(2,187)=1.820,*p*=.165 | | | | *F*(3,186)=1.832, *p*=.143 | | | |
| △*R ²* | .007 | | | | .012 | | | | .01 | | | |
| △*F* | *F*(1,188)=1.343, *p*=.248 | | | | *F*(1,187)=2.287,*p*=.132 | | | | *F*(1,186)=1.839, *p*=.177 | | | |

*Note.* BPL = benevolent leadership; Dependent variable: satisfaction; ***p* < .01; **p* < .05
