# Supplementary material for: Effect of paternalistic leadership on Chinese youth elite athletes’ satisfaction: Resilience as a moderator
Source: Front Psychol. 2022 Sep 29;13:1008163. doi: 10.3389/fpsyg.2022.1008163 (PMC9557739; doi:10.3389/fpsyg.2022.1008163)
Supplement: Supplementary file 4 [file Table_4.DOCX]

Supplementary Table 4

*Simple Slope Analysis*

| Manipulated variable level | Regression coefficients | Standard error | *t* | *p* | 95% CI | |
| --- | --- | --- | --- | --- | --- | --- |
| Mean | .021 | .095 | .22 | .826 | -.165 | .207 |
| High（+1SD） | -.15 | .133 | -1.124 | .262 | -.411 | .111 |
| Low（-1SD） | .192 | .109 | 1.763 | .08 | -.021 | .405 |
